# Effect of Prior Boriding on Microstructure and Mechanical Properties of Nanobainitic X37CrMoV5-1 Hot-Work Tool Steel

**DOI:** 10.3390/ma16124237

**Published:** 2023-06-07

**Authors:** Grzegorz Łukaszewicz, Michał Tacikowski, Michał Kulka, Krzysztof Chmielarz, Monika Węsierska-Hinca, Wiesław A. Świątnicki

**Affiliations:** 1 Faculty of Materials Science and Engineering, Warsaw University of Technology, ul. Wołoska 141, 02-507 Warsaw, Poland; michal.tacikowski@pw.edu.pl (M.T.); krzysztof.chmielarz.dokt@pw.edu.pl (K.C.); monika.wesierska.dokt@pw.edu.pl (M.W.-H.); wieslaw.swiatnicki@pw.edu.pl (W.A.Ś.); 2 Faculty of Materials Engineering and Technical Physics, Poznan University of Technology, Pl. M. Skłodowskiej-Curie 5, 60-965 Poznan, Poland; michal.kulka@put.poznan.pl

**Keywords:** hybrid treatment, pack boriding, nanobainite, microstructure, mechanical properties, dilatometry

## Abstract

The influence of prior pack boriding on the microstructure and properties of nanobainitised X37CrMoV5-1 hot-work tool steel was investigated in the present work. Pack boriding was conducted at 950 °C for 4 h. Nanobainitising consisted of two-step isothermal quenching at 320 °C for 1 h, followed by annealing at 260 °C for 18 h. A combination of boriding with nanobainitising constituted a new hybrid treatment. The obtained material exhibited a hard borided layer (up to 1822 ± 226 HV0.05) and a strong (rupture strength 1233 ± 41 MPa) nanobainitic core. However, the presence of a borided layer decreased mechanical properties under tensile and impact load conditions (total elongation decreased by 95% and impact toughness by 92%). Compared with borided and conventionally quenched and tempered steel, the hybrid–treated material retained higher plasticity (total elongation higher by 80%) and higher impact toughness (higher by 21%). It was found that the boriding led to the redistribution of carbon and silicon atoms between the borided layer and substrate, which could influence bainitic transformation in the transition zone. Furthermore, the thermal cycle in the boriding process also influenced the phase transformations during subsequent nanobainitising.

## 1. Introduction

Nanobainitic steels exhibit a combination of high strength and high plasticity [1,2,3], increased toughness [4,5], and good resistance to wear [6,7]. It seems that nanobainitic steels mitigate, to some extent, the conflict between high strength and plasticity, which is characteristic for conventionally heat-treated steels. This feature is due to their unique microstructure. It is built of dense interlocking bainite sheaves. Each sheave is formed of bainitic ferrite plates separated by thin layers of retained austenite, where the thickness of both does not exceed 100 nm [1,8]. Due to their favourable properties, parts and tools manufactured from nanobainitic steels are used in the mining [9], railway [10], forge [11], and military [12] industries. However, for some applications, their properties may not be sufficient. This refers to applications where, in addition to the excellent performance of the core, working conditions require a very high surface hardness and wear resistance, far exceeding the capabilities of nanobainitic steel.

One of the research directions aiming to overcome these limitations is the use of surface carburising treatment prior to the nanobainitising process. The works by Wasiluk et al. [13], Skołek et al. [14], and Wang et al. [15] should be mentioned here as examples of the effective application of combined carburising and nanobainitising. Hybrid treatments involving surface engineering also appear as a promising direction, expanding nanobainitic steels’ application areas. The effects of introducing carburising before nanobainitising have been studied. The effects of pre-boriding have still not been investigated.

Boriding is a thermochemical treatment leading to substrate saturation with boron atoms. Iron boriding has been known for nearly fourteen decades [16]. Steel boriding has been applied in the production of many tools and machine parts [17,18,19], e.g., dies, plungers, rolling bearings, and extruder screws. Boriding creates hard, wear- and corrosion-resistant layers [20,21]. Many different methods of boriding have been developed. The most popular are pack and paste boriding. In the case of pack boriding, the element is placed in a container filled with a powder (boriding medium), heated to a temperature of 800–1050 °C, and held for 0.25–30 h [18]. Conventionally, the heat treatment of borided steels includes quenching and tempering or isothermal quenching [18,22]. Post-boriding treatments can be carried out as separate treatments or performed in an integrated process. In the second case, austenitising of steel can be carried out simultaneously with boriding.

The heat treatment of borided steels has not been a frequently discussed topic until now. Most of the research to date concerns the optimisation of the boriding process or the characterisation of the produced layers. In the case of isothermal quenching, only a few works dealing with this issue can be indicated [23,24,25]. These are relatively new works, breaking the previous trend. Additionally, in these studies, attention was paid to the surface’s properties without investigating the core performance. Moreover, although bainitic transformation was used in these experiments, a nanobainitic microstructure was not achieved. The present research aims to provide missing knowledge in this field. The influence of prior boriding on the mechanical properties of nanobainitic X37CrMoV5-1 tool steel is presented and discussed here. The results achieved through the new hybrid treatment are compared to those achieved by conventional pack boriding and subsequent quenching and tempering.

## 2. Materials and Methods

In the present work, an EN X37CrMoV5-1 hot--work tool steel was used. Its chemical composition is presented in Table 1. Due to its chemical composition, this steel is suitable for nanobainitising [2].

**Figure 1:**
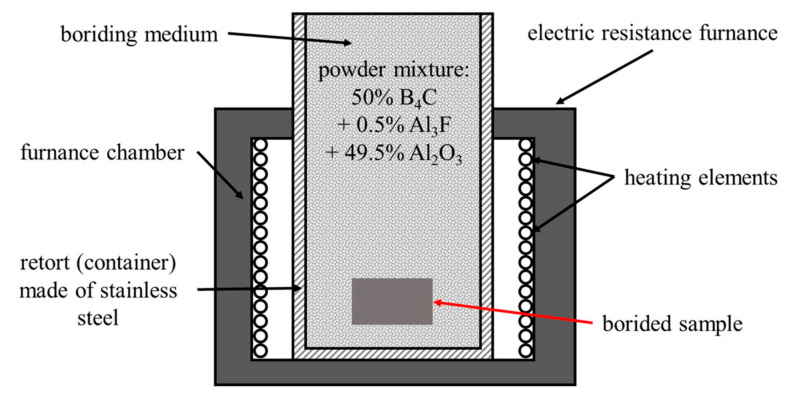
Scheme of pack boriding using open retort placed in a typical electric furnace.

The pack boriding process was carried out at 950 °C for 4 h. A powder mixture of 50% B_4_C (as a boron source), 0.5% AlF_3_ (as an activator), and 49.5% Al_2_O_3_ (as a diluent) was used as a boriding medium. The pack boriding was performed in an open retort filled with boriding powder and placed in a conventional electric furnace without an additional seal, as shown in the scheme in Figure 1. This boriding technique is described in the monograph [27] in detail. The retort was placed inside the furnace chamber in such a way that the upper part of the retort extended outside the furnace. The boriding powder in the upper part of the retort (outside the furnace) became its natural seal. The temperature of the surface of the boriding mixture was so low that this powder mixture did not oxidise. The gases created in the bottom part of the retort re-sublimated in its cold upper part and, therefore, did not escape the retort. After the process, the retort with the samples in the boriding medium was removed from the furnace chamber and cooled in the air. The samples were removed from the retort after it had cooled to ambient temperature to avoid oxidation.

**Figure 2:**
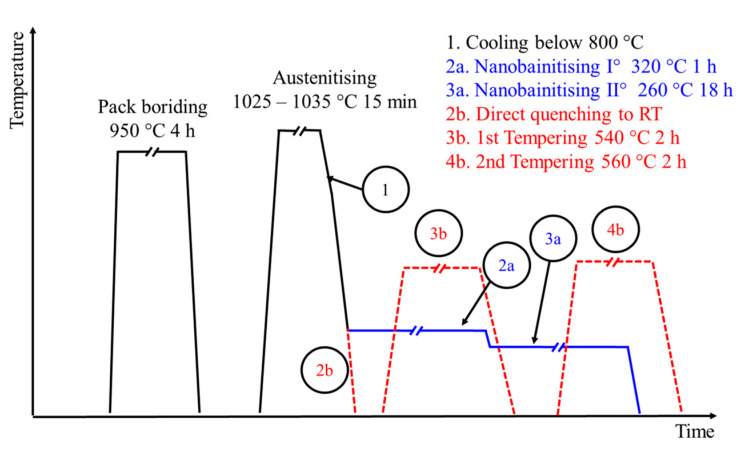
Scheme of hybrid heat treatments: boriding with nanobainitising and boriding with quenching and tempering.

Borided samples were subjected to two different heat treatments: nanobainitising (Br-NB) or quenching and tempering (Br-QT). In both cases, the austenitising was carried out similarly. The samples were treated in a vacuum tube furnace. Firstly, samples were heated in a furnace to austenitising temperature. After the batch reached a temperature of 1025 °C, the samples were heated for 15 min to about 1035 °C. Then, samples were moved to the cold zone of the furnace, where they were cooled to a temperature of 800 °C. Afterwards, they were transferred to subsequent heat treatment segments. This course of austenitising in a vacuum was necessary to prevent the oxidation of the borided layer at temperatures above 800 °C. In the first variant (hereinafter called Br-NB), samples after austenitising were nanobainitised through two-step isothermal quenching. During the first step, samples were held at 320 °C for 1 h. This segment allowed an amount of austenite to transform into bainitic ferrite and reduced the martensitic transformation start temperature M_s_ of the untransformed austenite below the temperature of the second step, fixed at 260 °C. Then, the second step of nanobainitising was conducted at 260 °C for 18 h. Samples after nanobainitising were cooled in the air. In the case of the second variant (hereinafter called Br-QT), the samples after austenitising were directly quenched in oil, tempered at 540 °C for 2 h and again at 560 °C for 2 h, and then cooled in the air. The batch temperature during the heat treatments was measured using the control sample with a thermocouple inside. The time of the isothermal segments was counted from when the control sample reached a temperature of 5 °C higher (nanobainitising) or lower (tempering) than the nominal temperature. The temperature regime of these segments was ± 5 °C. Additionally, the unborided samples were subjected to the same treatments (NB and QT variants). The variants of the treatments described in this work are summarised in Table 2 and shown schematically in Figure 2.

Dilatometric experiments were carried out using a DIL 805 L quenching dilatometer. Cylindrical samples with a diameter of ca. 2.8 mm and a length of approx. 10 mm were subjected to testing. The tests were carried out under a vacuum. Inert gas (helium) was used as a cooling agent.

Light microscopy and SEM observations were performed on the cylindrical samples’ cross sections. After mechanical grinding and polishing, chemical etching with Mi19Fe reagent was conducted to reveal the microstructure. The thickness of the microstructural zones (borides, porous) in the borided layer was determined based on an average of 30 measurements. SEM images of the microstructure were obtained using SEM Hitachi SU8000 in secondary electron mode (SE).

TEM observations were performed using the samples cut from the borided samples cores using wire electric discharge machining. Then, they were cut into thin slices, ground to a thickness of about 100 μm, and electrolytically thinned. Observations were carried out with the transmission electron microscope TEM JEOL 1200 EX II. The thicknesses of the bainitic ferrite plates and austenite layers were determined based on measurements from 30 randomly selected places. The obtained values were divided by π/2, according to the methodology described in the work of Garcia-Mateo et al. [28]. 

The phase composition of the layer of the borided disc was determined using X-ray diffraction (XRD). Measurements were carried out on a Rigaku SmartLab 3 kW diffractometer with the Cu tube radiation source and operating parameters of U = 40 kV and I = 30 mA. Bragg–Brentano θ/2θ measuring geometry was used with a measuring step of 0.02°.

To determine the concentration profile of the alloying elements across the borided layer, the glow-discharge optical emission spectroscopy (GDOES) technique was used. The examination was performed on a borided disc with a LECO GDS 850a spectrometer.

Magnetic tests were performed to determine the amount of retained austenite in the steel core after heat treatments. This method uses differences in the magnetic properties of phases existing in steel (ferromagnetic ferrite and paramagnetic austenite). During the examination, saturation magnetisation for the test material and the standard were compared. Samples in the form of round slices (approx. 1 mm thick, with a diameter of approx. 2.8 mm) obtained from borided samples’ cores were used.

Vickers HV0.05 microhardness measurements were performed on mechanically ground and polished cross sections of the borided cylindrical samples using a Future-Tech FM-810 tester. The indent size was measured using a Keyence VHX 7000 light microscope. Each value in the hardness distribution graph is the average of the 3 measurements. The hardness of the core is an average of 9 measurements. 

A static tensile test was conducted on the cylindrical samples using an MTS810 servo-hydraulic machine (MTS Systems, 100 kN load cell, Eden Prairie, MN, USA). The test was carried out with a constant elongation rate of 0.036 mm/s. An MTS extensometer with a gauge length of 25 mm was used to measure the elongation of the sample. For each treatment variant examination, 3 samples were tested, and the results were averaged.

Impact toughness tests were performed on the standard 10 × 10 × 55 mm rectangular samples (U-shaped notch) with a Charpy Zwick hammer (initial hammer energy of 300 J). For each treatment variant examination, 3 samples were tested, and the results were averaged.

## 3. Results

### 3.1. Microstructure after Boriding and after Hybrid Treatments

**Figure 3:**
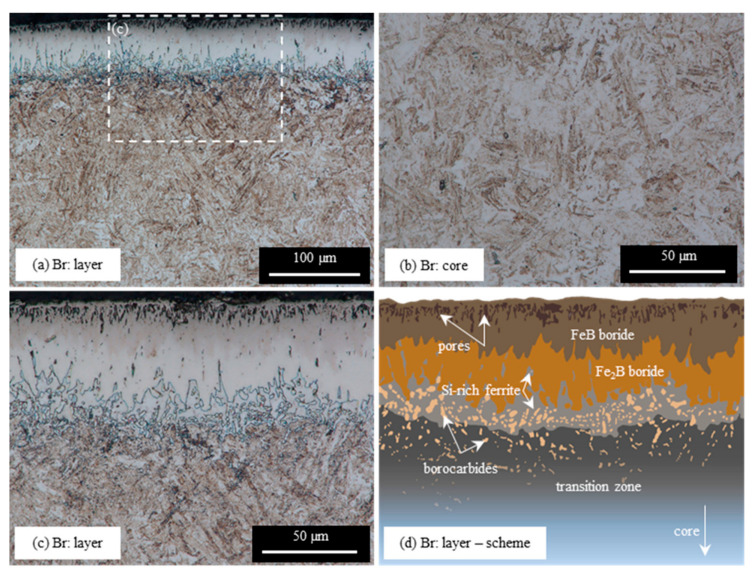
Microstructure of borided layer (**a**,**b**) and core (**c**) of borided EN X37CrMoV5-1 steel (Br variant) and scheme of borided steel surface (**d**).

**Figure 4:**
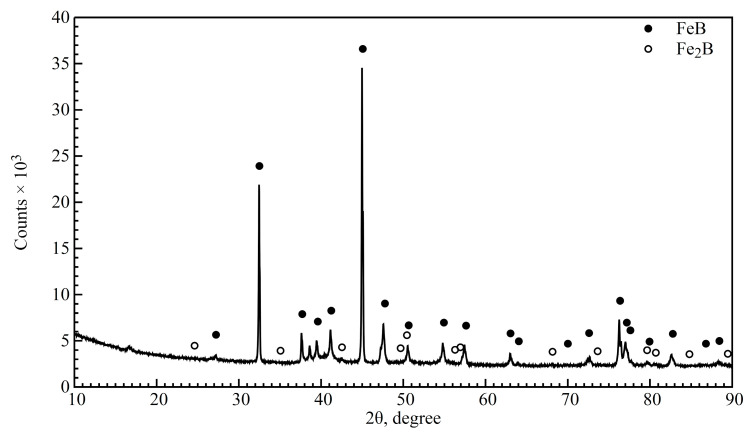
X-ray diffraction patterns of borided EN X37CrMoV5-1 steel surface (Br variant).

The microstructure of the layer produced on EN X37CrMoV5-1 steel by boriding at temp. 950 °C for 4 h (Br variant) is shown in Figure 3. The XRD analysis indicates the presence of two types of iron borides on the borided surface: FeB and Fe_2_B (Figure 4). The signal from the FeB phase is intensive, while the signal from the Fe_2_B phase is relatively weak. It should be mentioned that the high chromium content in steel could lead to the incorporation of this element in FeB- or Fe2B-type borides or the development of a small amount of chromium borides. Some studies have reported that during the boriding of steel, the Fe_2_B phase is formed first, and the FeB phase is formed by the accumulation of boron atoms in the layer (the diffusion of boron through Fe_2_B is strongly hindered) [29]. Hence, an increasing amount of Fe_2_B with increasing depth below the surface can be expected. In this work, the thickness of the borided layer (measured to the tips of the borided teeth) was 42.6 ± 8.3 µm. As can be seen in Figure 3, the borided layer exhibits saw-tooth morphology and is not physically homogenous. The borided layer consists of two boride types: FeB and Fe_2_B. The weak contrast between the FeB borides (dark pink) and Fe_2_B (light pink) indicates the upper zone of the FeB borides, up to a depth of 32.8 ± 7.2 µm.

In the borided layer closest to the surface, the porous zone forms (Figure 3a,c,d) and falls to a depth of 11.1 ± 2.2 µm. The near-surface porosity of borided steel is a well-known phenomenon reported by other researchers [30,31,32]. Porosity is commonly explained by the Kirkendall effect [33,34]. Below this zone, the borided layer is compact, although small objects with irregular shapes can be observed. These are fragments of silicon-rich ferrite areas intersected by the cross section or cavities after silicon-rich ferrite is chipped out. Silicon-rich ferrite is clearly visible between the layer of borides and the substrate, and its areas separate boride teeth. This phase is a typical component of borided steel layers containing more than 0.8 wt% silicon [18,19]. The interface between the layer and the substrate is relatively smooth. The substrate has many white, mostly globular precipitates just below the layer in the transition zone. The works of other researchers indicate that these are precipitates of borocarbides [19,35]. The amount of these precipitates decreases with the distance from the layer–substrate interface. The transition zone under the silicon-rich ferrite sublayer is not clearly visible (Figure 3c). However, more strongly etched areas and precipitates can be seen there (Figure 3c). The difference in etching indicates a different chemical composition of this area compared to the core. The microstructure of the core is dominated by martensite, formed from cooled austenite (Figure 3b). The local presence of bainite from continuous cooling is also possible.

**Figure 5:**
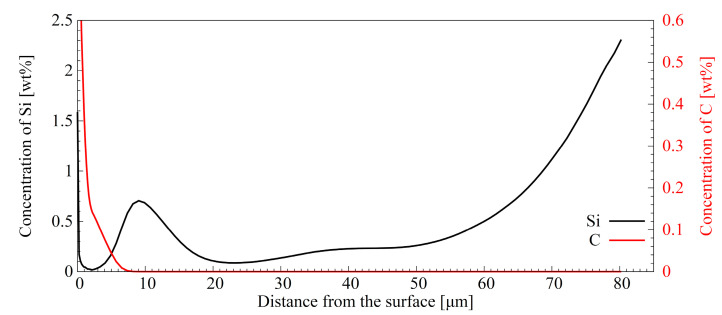
Distribution of silicon and carbon in borided layer of EN X37CrMoV5-1 steel (Br variant).

Figure 5 shows the GDOES profiles of silicon and carbon in the borided layer (Br variant). The concentration of carbon in the borided layer is close to zero. Additionally, a substantial redistribution of silicon atoms can be seen towards the layers. In the investigated steel, the amount of silicon is 1.16 wt%. (Table 1). The contribution of this element in the borided layer is in the range of ca. 0.1–0.7 wt%. Below a depth of 50 µm, the amount of silicon increases continuously, reaching a level of 2.3 wt%—twice of that in steel (Table 1). A higher contribution of silicon is associated with the presence of silicon-rich ferrite in this area (Figure 3).

**Figure 6:**
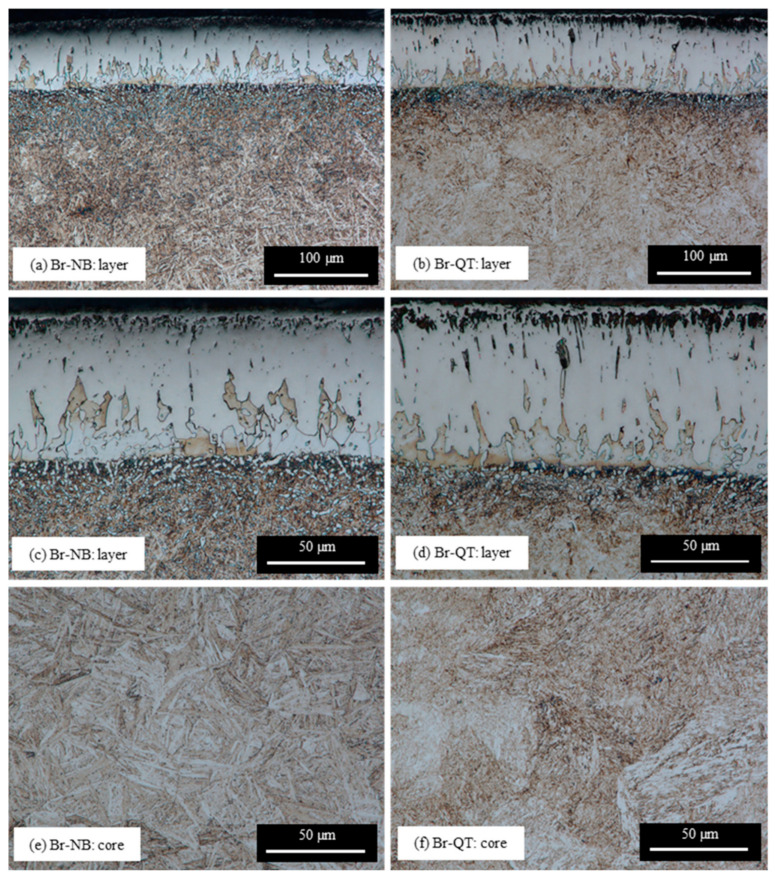
Microstructures after hybrid treatments of EN X37CrMoV5-1 steel: Br-NB layer (**a**,**c**) and core (**e**), Br-QT layer (**b**,**d**) and core (**f**).

**Figure 7:**
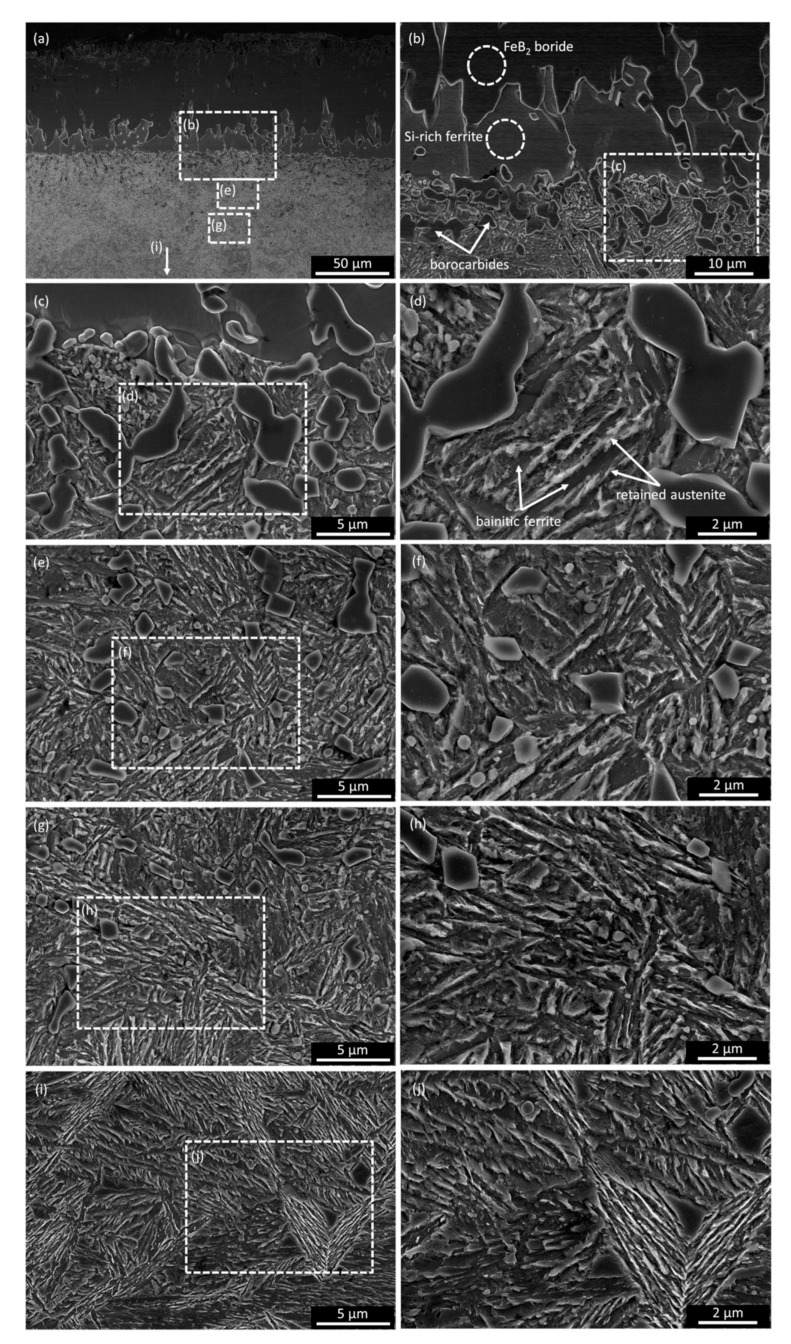
SEM microstructures of EN X37CrMoV5-1 steel core after the variant Br-NB heat treatment, showing the gradient microstructure obtained after hybrid treatment: layer and transition zone (**a**–**c**), transition zone (**d**–**h**), core (**i**,**j**).

The microstructures of the layer, the transition zone, and the core after both hybrid treatment variants (Br-NB and Br-QT) of EN X37CrMoV5-1 steel are shown in Figure 6. As can be seen, the substrate area is transformed after post-boriding treatments. In the case of post-boriding nanobainitising (Br-NB variant), heat treatment resulted in a microstructure composed of bainitic ferrite sheaves and retained austenite (Figure 6a,c,e). According to magnetic measurements, the amount of retained austenite in the core after treatment was 33.2 ± 1.7 vol%. Since nanobainitising with two isothermal steps was used, two populations of bainitic ferrite sheaves can be distinguished in the material (Figure 6e). Larger sheaves were formed during the isothermal step at temp. 320 °C, and smaller ones formed during the subsequent step at temp. 260 °C. Bainitic ferrite sheaves interlock, leading to the intense refinement of retained austenite. It can also be observed that the bainitic ferrite is more refined near the layer (transition zone, Figure 6c). As can be seen, the globular precipitates that appeared below the layer during boriding remain after the post-boriding treatment. SEM observations provided more information about the microstructure of the nanobainitised steel below the borided layer (Figure 7). Precipitates of borocarbides are clearly visible (Figure 7b–h). They are much larger than fine precipitates of carbides (probably primary MC-type carbides, which were not dissolved during austenitising). The amount of borocarbides precipitates is significant in the zone below the silicon-rich ferrite sublayer. There are visible bridges connecting some of the globular precipitates, indicating partial coagulation. With the distance towards the core, the number and size of the precipitates decreases until they disappear in the core zone (where fine carbides are still visible—Figure 7i,j). The presence of precipitates affects the morphology of bainitic ferrite sheaves, limiting their length. It cannot be ruled out that the present precipitates were sites of heterogeneous nucleation for bainitic ferrite. However, it can be said with certainty that the borocarbides blocked the growth of bainitic ferrite sheaves. Interestingly, the closer the bainitic ferrite formed to the borided layer, the broader its plates became.

**Figure 8:**
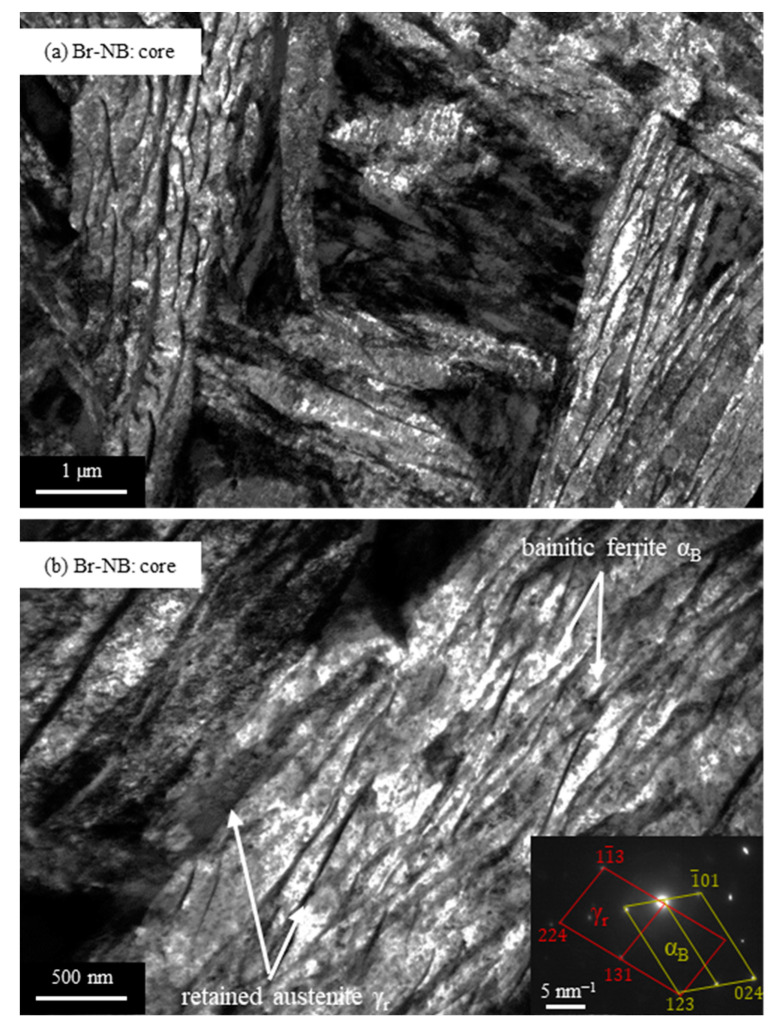
TEM microstructures of EN X37CrMoV5-1 steel core after the variant Br-NB heat treatment, showing the nanobainitic areas. Subfigures (**a**,**b**) represent different locations in the microstructure. For microstructure shown in (**a**) the corresponding diffraction pattern has been given.

TEM observations revealed the presence of nanobainitic areas in the steel core after Br-NB treatment (Figure 8). The average thickness of the bainitic ferrite plates was 70 ± 36 nm.

In the case of the Br-QT hybrid treatment variant, the microstructure of the EN X37CrMoV5-1 steel substrate and core is typical for the performed heat treatment. It consists of tempered martensitic, retained austenite, and carbides (Figure 6c,d,f). According to magnetic measurements, the amount of retained austenite in the core after the Br-QT treatment was 7.4 ± 2.1 vol%. Moreover, the changes in the substrate’s microstructure in the vicinity of the borided layer are also present, as well as globular precipitatates retained after boriding.

### 3.2. Dilatometry

**Figure 9:**
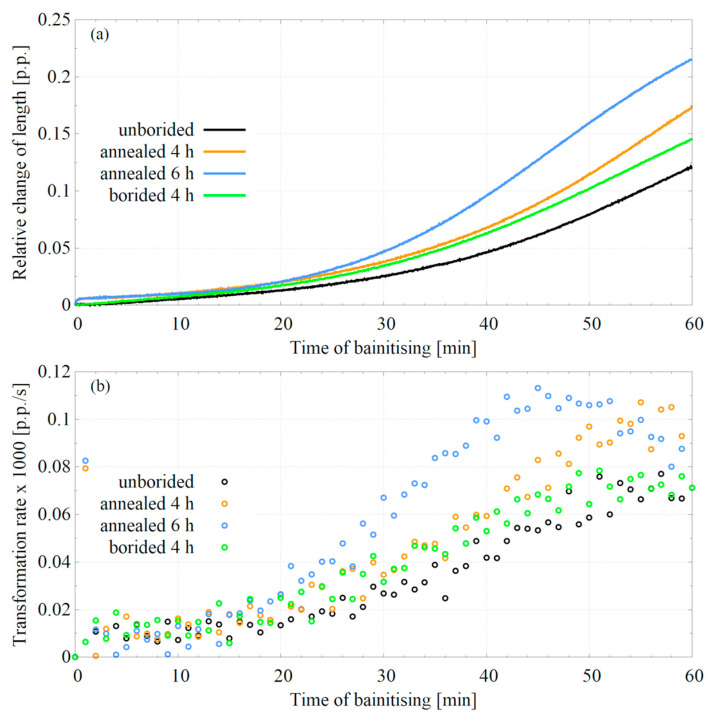
Dilatometric curves for nanobainitising segments of EN X37CrMoV5-1 steel at 320 °C (**a**) and corresponding bainitic transformation rate (**b**).

In order to determine the effect of prior boriding thermal cycle on phase transformations in steels during hybrid treatment, dilatometric studies were performed. They were conducted on samples obtained from the core of the borided specimen (‘borided 4 h’ variant) and unborided steel as a reference sample (‘unborided’ variant). In addition, two variants were carried out in which isothermal quenching was preceded by annealing, simulating boriding at 950 ℃ with different durations (‘annealed 4 h’ and ‘annealed 6 h’ variants). Figure 9a shows the dilatometric curves for the first step of bainitising at 320 °C, and Figure 9b shows the bainitic transformation rate. The curves lose their convergence after ca. 20 min of the segment—the bainitic transformation occurs more intensively in the previously borided sample. Comparing the bainitic transformation kinetics of unborided, 4 h borided, and 4 h annealed specimens, it can be seen that the thermal cycle of the previous treatment leads to the acceleration of the bainitic transformation. Moreover, extending the boriding time may intensify this effect.

### 3.3. Mechanical Properties

**Figure 10:**
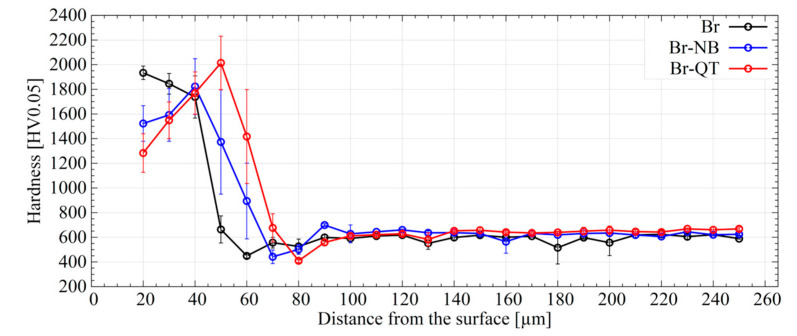
Microhardness distribution of the borided EN X37CrMoV5-1 steel for the different post-boriding treatment variants.

The results of the microhardness measurements of the borided layer and the substrate for the Br variant are shown in Figure 10 and in Table 3. The results for hybrid treatments (Br-NB and Br-QT variants) are also presented. As can be seen, boriding leads to the formation of a layer with a microhardness reaching almost 2000 HV0.05 (Br variant). It can be noticed that the hardness lowers as the borided layer changes within the depth. The steeper decrease in microhardness that occurs for depths greater than 40 µm from the specimen surface is caused by silicon-rich ferrite between the borided layer and the substrate (Figure 3c). This relatively soft phase [36] causes a drop in the hardness, the bottom of which falls on 60 µm (449 ± 18 HV0.05). At a depth of 60 µm, silicon-rich ferrite is the dominant phase (Figure 3c). At higher depths from the surface, the microhardness increases and is maintained in the range of 585–645 HV0.05. Subsequent nanobainitising reduces the hardness to ca. 20–40 µm below the surface (Br-NB variant), as opposed to the Br variant. This effect is probably due to the degradation of the porous zone as a result of the thermal stresses introduced during the post-boriding treatment. The effect of the lower hardness associated with the presence of silicon-rich ferrite is retained after nanobainitising. The hardness of the core is maintained in the range of 600–667 HV0.05. Quenching and tempering (Br-QT variant) as conventional treatment leads to similar results.

**Figure 11:**
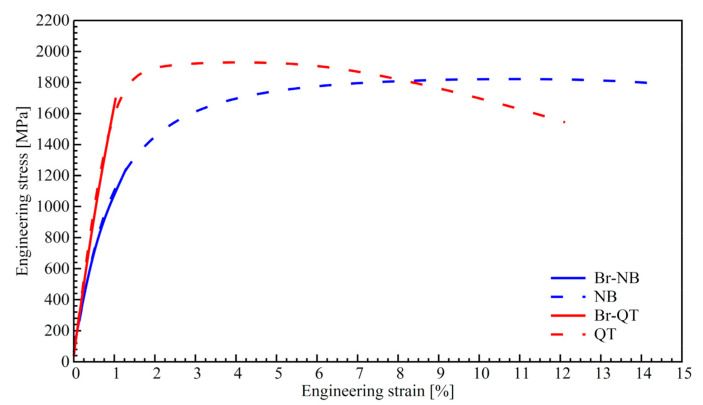
Tensile tests curves obtained for different treatment variants of EN X37CrMoV5-1 steel.

Figure 11 shows the results of the static tensile test of steel samples after treatments with prior boriding (Br-NB, Br-QT) and without it (Nb, QT). The mechanical properties determined in these tests are collected in Table 3. Unborided steel after nanobainitising (NB variant) reaches an ultimate tensile strength (UTS) of 1858 MPa, yield strength (YS) of 878 MPa, and uniform and total elongations of 9.84 and 14.41%, respectively. The steel after conventional treatment (QT variant) reaches a UTS of 1938 MPa, YS of 1560 MPa, and uniform and total elongations, respectively, of 3.15 and 11.38%. The differences between the behaviour of differently heat-treated samples during the tensile test are due to their internal structure. In the case of the NB variant, the steel is composed of bainitic ferrite (including nanobainite) and retained austenite. In turn, in the case of the QT state, the steel is made of tempered martensite and retained austenite. As previously indicated, nanobainitising allows the preservation of more than 3–4 times greater amounts of retained austenite in the microstructure than quenching and tempering. Thus, the different microstructures of both variants lead to significant differences in the strength of the material and its behaviour under plastic deformation conditions. As one can notice, prior boriding weakens the material, leading to earlier rupture of the samples (variants Br-NB and Br-QT). Evidently, the curves for hybrid treatments coincide with their equivalents in the unborided states (Figure 11). However, with an engineering deformation of ca. 1.2%, the steel samples undergo a fracture. In the case of previously borided samples, material failure occurred outside the measuring range of a sample, i.e., between the measuring area and the threaded part.

The limit of engineering strain is similar for the Br-NB and Br-QT variants (Figure 11), indicating that the factor regulating the resistance of the material was the condition of the produced borided layer. After boriding and nanobainitising (Br-NB variant), steel reached a rupture strength (RS) of 1233 MPa, a YS of 842 MPa, and a total elongation of 0.74%. Borided steel after quenching and tempering (Br-QT variant) reached an RS of 1573 MPa, a YS of 1435 MPa, and a total elongation of 0.41%. It is worth noting that in the case of hybrid treatments, the yield strength reached slightly lower values than in the case of corresponding treatments without boriding. This may be a consequence of the influence of the heat cycle of boriding on the microstructure of steel before subsequent heat treatments and, thus, on the phase transformations occurring during them.

The results of the impact tests after the treatments are shown in Table 4. As can be seen, the steel after the nanobainitising shows a high impact toughness at the level of 41.8 J/cm^2^. It is 60% higher than the impact toughness of steel after quenching and tempering (26.1 J/cm^2^). Nanobainitic steel’s advantage is its resistance to crack propagation microstructure. Nanobainitic microstructure contains interlocking bainitic ferrite sheaves composed of nanoplates and a significant proportion of ductile retained austenite, which counteract the propagation of cracks. Prior boriding leads to a significant reduction in the impact toughness of the material. In the case of hybrid treatment with nanobainitising (Br-NB), the impact toughness was 3.5 J/cm^2^ (reduction of 92%), and with quenching and tempering (Br-QT) it was 2.9 J/cm^2^ (reduction of 89%). However, the reduction in impact toughness was higher for the Br-NB variant; it was higher than that of the Br-QT variant by 21%.

## 4. Discussion

The combination of boriding and subsequent nanobainitising hybrid treatment presents interesting perspectives for practical applications. From the perspective of nanobainitic steels, the advantage is increasing hardness and wear resistance. Understandably, material’s overall properties are not a simple sum of the properties of its components. They are determined by the mutual influence of the layer and the substrate. This relationship includes both the advantages and disadvantages of the system’s components. Therefore, it is crucial to identify the interaction between the different processes and consider the possible consequences.

### 4.1. Influence of Borided Layer on Mechanical Properties

The noticeable and most radical change caused by prior boriding is the formation of a borides layer. In this work, the borided layer consisted of FeB- and Fe_2_B-type borides with a thickness of 42.6 ± 8.3 µm (Figure 3). The presence of the borided layer caused an increase in microhardness, even up to ca. 2000 HV0.05 (Figure 10). This hardness change corresponds to more than a three-fold increase compared to the hardness of the core. The borided layer microstructure was cognate with that obtained by other researchers as a result of the boriding process on similar types of steel [30,31,37,38,39]. The relatively large amount of chromium in steel certainly favours the presence of FeB-type borides. On the other hand, chromium leads to smoothing of the interface between the layer and the substrate [19]. Based on the work of other researchers, the occurrence of small amounts of chromium borides [30,40,41] and the incorporation of chromium in the iron boride lattice can be expected [42]. Krelling et al. [37] and Morón et al. [39] reported increased wear resistance of similar hot-work tool steel due to the boriding. High resistance to wear is a well-known feature of borided layers and will not be discussed in this paper. Understandably, through the proper selection of the boriding method, process parameters, and the chemical composition of steel, the structure and properties of borided layers can be tailored [29]. It should also be noted that the subsequent nanobainitising did not cause significant changes in the borided layer microstructure (Figure 6 and Figure 7) and microhardness distribution (Figure 10). 

In addition to the increase in surface microhardness and the expected increase in resistance to wear, the presence of the borided layer caused a decrease in the tensile strength and ductility of the entire system. It is known that layers of borides are hard but also brittle [29]. A layer of borides on steel’s surface increases its compressive strength but significantly reduces its tensile strength. According to Krukovich et al., a layer of borides diminishes the ultimate strength by 5–20%, relative elongation by 20–50%, and impact strength by 1.5–2 times [29]. In the present study, the borided and nanobainitised steel decreased in strength (UTS vs. RS) by 34%, in total elongation by 95%, and in impact toughness by 92%. This relatively severe degradation of mechanical properties is due mainly to non-optimal boriding conditions. The brittleness of the borided layer manifests itself as a factor regulating the fragility of the entire system. It can be reduced by preventing the formation of FeB-type borides, eliminating porosity, and adjusting the thickness of the borided layer [29]. Such improvement can be achieved by optimising the boriding conditions. It should be underlined that prior boriding led to increased brittleness of both nanobainitised and conventionally quenched and tempered steels. For the Br-QT variant, prior boriding reduced strength (UTS vs. RS) by 19%, total elongation by 96%, and impact toughness by 89%. These values do not mean that the quenched and tempered steel is more resistant to the effect of prior boriding. The scale of properties’ deterioration depends, on the one hand, on the base core properties (compare with unborided variants NB and QT in Table 4) and on the crack propensity of the borided layer on the other. With reduced layer fragility, the benefits of the nanobainitic core should be enhanced.

### 4.2. Redistribution of Alloying Elements

Another effect associated with prior boriding is the redistribution of alloying elements (Figure 5). This effect is due to their limited solubility in the borides’ crystal network. Litoria et al., in their research, included a broad compositional analysis of a pack-borided layer developed on 34CrAlMo5-10 steel [35]. They found that carbon, aluminium, and silicon solubilities were almost zero in the borided layer. The atoms of these elements were pushed into the core areas under the layer. Substantial redistribution also affected chromium, manganese, molybdenum, and nickel. The redistribution of carbon atoms through a borided layer was also shown by Liu et al. [24] and Campos-Silva et al. [43].

In this work, the concentration of carbon in the analysed area was decreased from 0.37 wt%, corresponding to the unborided materials (Table 1), to a level close to zero (Figure 5). Such a substantial depletion in carbon atoms of this layer area is associated with their pushing towards the substrate. A visible consequence of this redistribution is the formation of precipitates known as borocarbides [19,35]. The globular precipitates of this phase are visible in the microstructure after boriding (Figure 3c) and after subsequent nanobainitising or quenching and tempering (Figure 6 and Figure 7). Apart from the influence of borocarbides on the mechanical properties of borided steel, one aspect should be considered: the influence on the kinetics of bainitic transformation and the formation of acicular ferrite. Acicular ferrite is a structural component made up of ferritic products (including bainitic ferrite) nucleating inside austenite grains on non-metallic inclusions or precipitates and leading to star-like morphology [44]. If acicular ferrite nucleation occurs, the bainitic transformation is accelerated, which leads to a highly refined microstructure with highly interlocking components. As studies by other researchers show, not all precipitates are effective for the formation of acicular ferrite [44]. Therefore, there is a need for verification if borocarbides can promote the formation of acicular ferrite. Apart from the question of borocarbides, the enrichment of the substrate in carbon can cause significant changes in bainitic transformation. With the increasing amount of carbon, undercooled austenite shows decreased susceptibility to bainitic transformation [45]. On the other hand, the solution strengthening helps reduce the thickness of the resulting bainitic ferrite plates [46]. The influence of carbon concentration on the size of bainitic transformation products was deliberately used by Wasiluk et al. [13] and Skołek et al. [14] to obtain nanobainite in carburised steels. However, in our studies, we observed the coarsening effect of the bainitic ferrite plates in the transition zone of the substrate (Figure 7).

Attention should also be paid to the redistribution of silicon and the observed formation of the silicon-rich ferrite zone. This effect is known for steels with a silicon fraction higher than 0.8 wt%. [18,19]. Since silicon solubility in the borides’ crystal lattice is very low, its atoms are pushed out by the growing borides layer. Redistribution leads to silicon accumulation under the borided layer and the formation of the silicon-rich ferrite area. Silicon-rich ferrite is an undesirable component of the borided layers. Using steel with a lower silicon content would prevent this component’s formation. However, silicon is an essential element from the point of view of nanostructured steel by bainitic transformation. Since it is an insoluble atom in the carbides’ crystal lattice, it prevents their precipitation during bainitising [45,47]. Thus, the carbon from the newly-formed bainitic ferrite plates enriches the surrounding austenite instead of forming carbides. However, the sublayer of silicon-rich ferrite did not undergo a bainitic transformation since it had not turned into austenite during austenitising.

### 4.3. Influence of Thermal Cycle

The last effect worth discussing is the influence of the thermal cycle itself. Depending on the method, boriding requires heat the steel at 550–1100 °C for 0.15–30 h [18,22]. The temperatures for powder boriding are in the upper range. Apart from the changes related to the development of the borided layer on the surface, the boriding process temperatures lead to phase transformations in the processed material’s core. Consequently, the base materials before the nanobainitising process differ if prior boriding is applied. This leads to changes in the kinetics of phase transformations occurring during nanobainitising, as demonstrated in the present work (Figure 9). In contrast to the redistribution of alloying elements pushed by the borides layer, the effect of the thermal cycle covers the entire volume of the treated material. This effect should be considered when designing hybrid heat treatments. Because, in this work, the effect of the thermal cycle was considered, the nanobainite was effectively obtained.

The change in the bainitic transformation kinetics is essential not only from the technological point of view (the possibility of shortening the heat treatment time). Due to the autocatalytic nature of the bainitic transformation, it should be expected that the change in its kinetics affects the spatial organisation of the bainitic ferrite sheaves. Since the kinetics change also influences the carbon atoms’ emission intensity to the untransformed retained austenite, the changes may also affect the stabilisation of the retained austenite. Therefore, when designing a hybrid heat treatment, it is necessary to consider the influence of the thermal cycle on the phase transformations’ kinetics. Not only the fraction of bainitic ferrite but also the fraction of retained austenite and the morphologies of both components are significant for mechanical properties. Bainitic ferrite is essential for material strength, while retained austenite is critical for toughness.

## 5. Conclusions

1. Applying pack boriding prior to the nanobainitising allowed obtaining a new material based on the X37CrMoV5-1 hot-work tool steel. The main effect caused by the hybrid treatment was the formation of a hard saw-tooth-like layer of borides with a thickness of ca. 43 µm. The layer was composed of FeB (closer to the surface) and Fe_2_B (located in deeper areas of the layer) borides. Due to the borided layer’s formation, the microhardness of the steel surface increased from ca. 600 HV0.05 (in the nanobainitic core) up to 1800 HV0.05.
2. Besides a significant increase in hardness, the prior boriding process decreased other mechanical properties of nanobainitised steel (Br-NB) compared to the unborided state (NB). For the nanobainitised state without boriding, the ultimate tensile strength was 1858 MPa, the yield strength was 878 MPa, the uniform elongation was 9.84%, the total elongation was 14.41%, and the impact toughness was 41.8 J/cm^2^. In the case of the previously borided and nanobainitised state, the mechanical properties were, respectively, rupture strength of 1233 MPa, yield strength of 842 MPa, total elongation of 0.74% (there was no uniform elongation), and impact toughness of 3.5 J/cm^2^. The reason for the decrease in the mechanical properties’ values is the brittleness of the borided layer. Additionally, borided steel after quenching and tempering (Br-QT) exhibited a decrease in these mechanical properties. The Br-NB state retained higher plasticity and impact toughness than the Br-QT state, indicating the advantage of the new hybrid treatment over the conventional treatment. This property indicates that the nanobainitic core is less susceptible to the propagation of cracks formed in the borided layer.
3. The formation and growth of the borided layer during the pack boriding led to the redistribution of alloying elements in X37CrMoV5-1 steel. Significantly, the silicon and carbon atoms were pushed out of the boride zone towards the substrate due to their strongly limited solubility in borides. In the case of silicon atoms, this led to the formation of the soft silicon-rich ferrite zone. In the case of carbon atoms, this resulted in the precipitation of borocarbides in the transition zone. An intense refinement in the bainitic structure was observed in the transition zone formed between the borided layer and the nanobainitic core.
4. Dilatometric studies have shown that the thermal cycle of prior boriding led to a change in the kinetics of phase transformations during the nanobainitising process. These changes included the acceleration of the kinetics of bainitic transformation during isothermal hold at temp. 320 °C. This effect should be taken into account when designing hybrid heat treatments. However, as it was established experimentally, observed changes did not prevent the formation of nanobainite in the borided steel.

## Figures and Tables

**Table 1:** Chemical composition of EN X37CrMoV5-1 steel used in this work and compositional range according to EN ISO 4957 standard [26] (in wt%).

Element	C	Si	Cr	Mn	Mo	Ni	V	Fe and Impurities
This work	0.37 ± 0	1.16 ± 0.01	4.95 ± 0.05	0.43 ± 0	1.22 ± 0.01	0.26 ± 0	0.40 ± 0	Balance
Standard	0.33–0.41	0.80–1.20	4.80–5.50	0.25–0.50	1.10–1.50	-	0.30–0.50	Balance

**Table 2:** Parameters and designations of the different heat treatment variants used in this work.

Designation	Boriding	Austenitising	Quenching	Tempering
Br	950 °C 4 h	-	-	-
Br-NB		1025–1035 °C 15 min	isothermal 320 °C 1 h + 260 °C 18 h	-
Br-QT			direct to RT	540 °C 2 h + 560 °C 2 h
NB	-		isothermal 320 °C 1 h + 260 °C 18 h	-
QT	-		direct to RT	540 °C 2 h + 560 °C 2 h

**Table 3:** Microhardness distribution of the borided EN X37CrMoV5-1 steel for the different post-boriding treatment variants.

Distance from the Surface [µm]	Br	Br-NB	Br-QT
20	1934 ± 54	1523 ± 144	1283 ± 156
40	1738 ± 171	1822 ± 226	1770 ± 171
50	663 ± 110	1373 ± 423	2014 ± 218
60	449 ± 18	894 ± 307	1417 ± 381
80	526 ± 60	502 ± 39	410 ± 15
100	591 ± 3	628 ± 73	611 ± 16

**Table 4:** Mechanical properties obtained for the different treatment variants. The ultimate tensile strength (UTS) shown is for unborided variants, and the rupture strength (RS) shown is for borided variants.

Variant	UTS or RS [MPa]	Yield Strength R_p0.2_ [MPa]	Uniform Elongation [%]	Total Elongation [%]	Impact Toughness KU [J/cm^2^]
NB	1858 ± 31	878 ± 17	9.84 ± 0.36	14.41 ± 1.62	41.8 ± 2.4
QT	1938 ± 6	1560 ± 13	3.15 ± 0.24	11.38 ± 0.33	26.1 ± 2.4
Br-NB	1233 ± 41	842 ± 13	-	0.74 ± 0.03	3.5 ± 0.2
Br-QT	1573 ± 101	1435 ± 30	-	0.41 ± 0.41	2.9 ± 0.5

## Data Availability

All data included in this study are available upon request by contacting the corresponding author.
